# The role of Effortful Control and Executive Attention in Mood Disorders

**DOI:** 10.1192/j.eurpsy.2022.174

**Published:** 2022-09-01

**Authors:** P. Ossola

**Affiliations:** University of Parma, Department Of Medicine And Surgery, Parma, Italy

**Keywords:** Depression, computational psychiatry, bipolar disorder, Effortful Control

## Abstract

In the first part of the talk I will show that using a computational approach, namely the Drift Diffusion Model, the efficiency of executive attention (EA) is associated with effortful control, the self-regulatory aspect of temperament in a sample of n=427 healthy subjects. Then I will demonstrate how, applying the same model, EA measured through the same conflict resolution task predicts longitudinally a sooner relapse in a sample of n=64 subjects with bipolar and depressive disorders. Lastly I will discuss how EA is associated with obsessive compulsive symptoms in the same clinical population and how they interact in predicting the course of illness. Altogether these results suggest that there is an association between self-reported effortful control and EA and that treatments targeting executive functions could be crucial in preventing relapses in subjects with mood disorders experiencing obsessive compulsive-symptoms.

**Disclosure:**

No significant relationships.

